# Supplementary figures and images for: Plant structural diversity alters sediment retention on and underneath herbaceous vegetation in a flume experiment
Source: PLoS One. 2021 Mar 18;16(3):e0248320. doi: 10.1371/journal.pone.0248320 (PMC7971462; doi:10.1371/journal.pone.0248320)

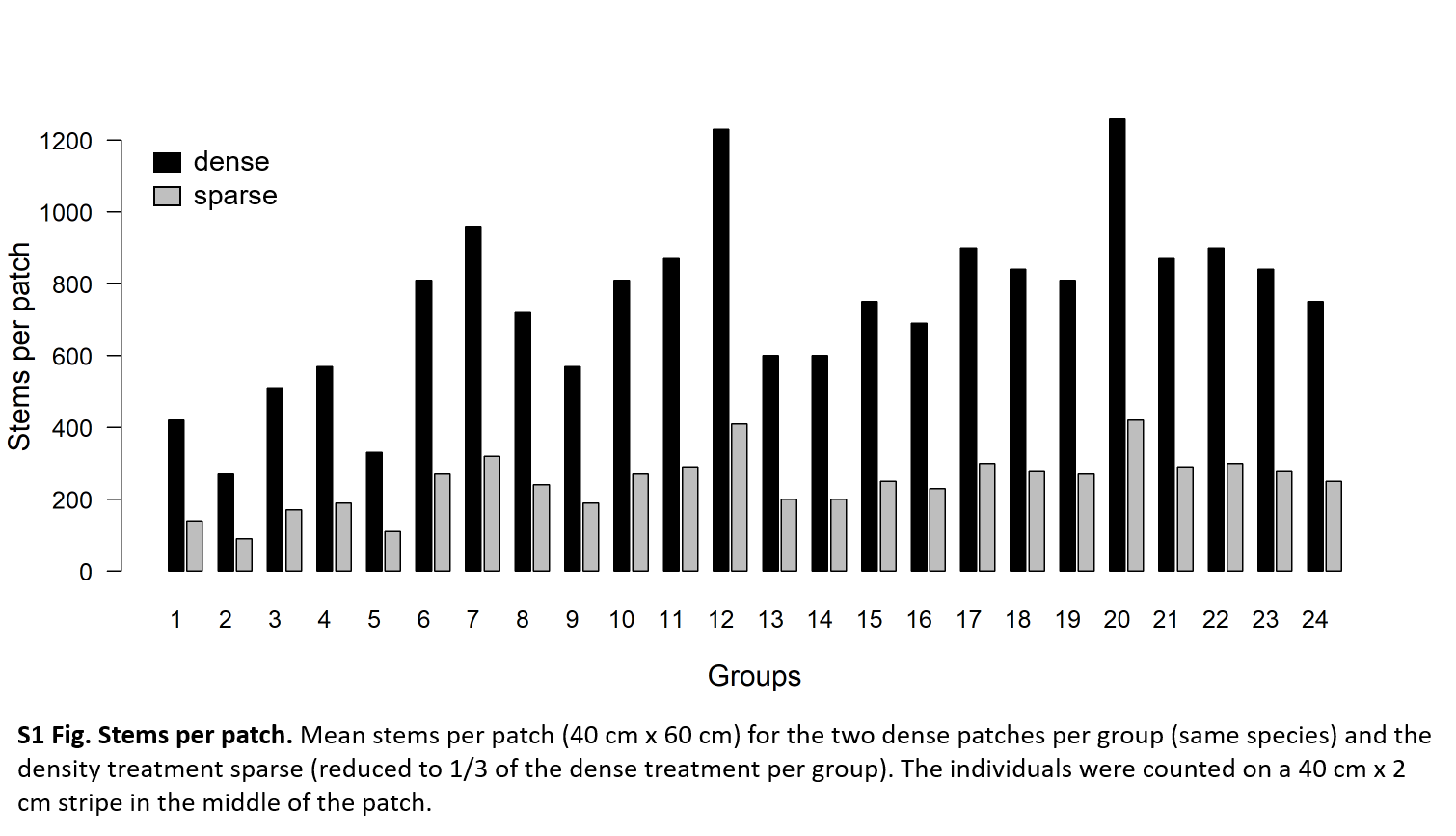

Supplement: S1 Fig — Mean stems per patch (40 cm x 60 cm) for the two dense patches per group (same species) and the density treatment sparse (reduced to 1/3 of the dense treatment per group). The individuals were counted on a 40 cm x 2 cm stripe in the middle of the patch. (TIF) [file pone.0248320.s001.tif]
