# Supplementary material for: Plant structural diversity alters sediment retention on and underneath herbaceous vegetation in a flume experiment
Source: PLoS One. 2021 Mar 18;16(3):e0248320. doi: 10.1371/journal.pone.0248320 (PMC7971462; doi:10.1371/journal.pone.0248320)
Supplement: S1 Table — Species selection of the experiment according to leaf pubescence, growth height and growth form. (DOCX) [file pone.0248320.s002.docx]

**S1 Table. Species selection.** Species selection of the experiment according to leaf pubescence, growth height and growth form.

| **Species** | **Leaf pubescence** | **Growth height** | **Growth form** |
| --- | --- | --- | --- |
| *Helictotrichon pratense* | high | small | grass |
| *Holcus lanatus* | high | small | grass |
| *Bromus hordeaceus* | high | small | grass |
| *Avena strigosa* | high | tall | grass |
| *Bromus inermis* | high | tall | grass |
| *Elymus repens* | high | tall | grass |
| *Potentilla argentea* | high | small | herb |
| *Silene noctiflora* | high | small | herb |
| *Myosotis stricta* | high | small | herb |
| *Artemisia vulgaris* | high | tall | herb |
| *Stachys palustris* | high | tall | herb |
| *Urtica dioica* | high | tall | herb |
| *Agrostis capillaris* | low | small | grass |
| *Festuca rubra* | low | small | grass |
| *Poa nemoralis* | low | small | grass |
| *Dactylis glomerata* | low | tall | grass |
| *Phalaris arundinacea* | low | tall | grass |
| *Phragmites australis* | low | tall | grass |
| *Glechoma hederacea* | low | small | herb |
| *Stellaria holostea* | low | small | herb |
| *Viola tricolor* | low | small | herb |
| *Lysimachia vulgaris* | low | tall | herb |
| *Rumex acetosa* | low | tall | herb |
| *Chenopodium album* | low | tall | herb |
