# Supplementary material for: Plant structural diversity alters sediment retention on and underneath herbaceous vegetation in a flume experiment
Source: PLoS One. 2021 Mar 18;16(3):e0248320. doi: 10.1371/journal.pone.0248320 (PMC7971462; doi:10.1371/journal.pone.0248320)
Supplement: S2 Table — Definition of the predictor variables calculated from the images. *Without a unit, since length was not scaled. (DOCX) [file pone.0248320.s003.docx]

**S2 Table. Predictor definitions.** Definition of the predictor variables calculated from the images. *Without a unit, since length was not scaled.

| **Predictor** | **Details** |
| --- | --- |
| Vertical density | Percent of vegetation pixels on the image of standard size |
| Mean height | Mean height of vegetation pixels on the image * |
| Median height | Median height of vegetation pixels on the image * |
| Height variation | Standard deviation of vegetation pixel height on the image * |
