# Supplementary material for: Plant structural diversity alters sediment retention on and underneath herbaceous vegetation in a flume experiment
Source: PLoS One. 2021 Mar 18;16(3):e0248320. doi: 10.1371/journal.pone.0248320 (PMC7971462; doi:10.1371/journal.pone.0248320)
Supplement: S3 Table — Residuals of sediment on the vegetation and underneath the vegetation explained by the measured variables. (DOCX) [file pone.0248320.s004.docx]

**S3 Table. Statistical model results.** Residuals of sediment on the vegetation and underneath the vegetation explained by the measured variables.

|  | Residuals of sediment on the vegetation | | | | |
| --- | --- | --- | --- | --- | --- |
|  | **Estimate** | **Std. Error** | **t value** | **Pr(>\|t\|)** | **Sig.** |
| **(Intercept)** | -1.50E-15 | 0.226 | 0.000 | 1.000 |  |
| **Log biomass** | 1.824 | 0.324 | 5.626 | 1.98E-07 | *** |
| **Vertical density** | -0.622 | 0.337 | -1.845 | 0.068 | . |
| **Height variation** | -0.520 | 0.241 | -2.157 | 0.034 | * |
|  |  |  |  |  |  |
|  | Residuals of sediment underneath the vegetation | | | | |
|  | **Estimate** | **Std. Error** | **t value** | **Pr(>\|t\|)** | **Sig.** |
| **(Intercept)** | 1.50E-15 | 0.091 | 0.000 | 1.000 |  |
| **Biomass** | 0.346 | 0.125 | 2.767 | 0.007 | ** |
| **Vertical density** | -0.331 | 0.125 | -2.647 | 0.010 | ** |
