# Supplementary material for: Plant structural diversity alters sediment retention on and underneath herbaceous vegetation in a flume experiment
Source: PLoS One. 2021 Mar 18;16(3):e0248320. doi: 10.1371/journal.pone.0248320 (PMC7971462; doi:10.1371/journal.pone.0248320)
Supplement: Attachment — Submitted filename: Response to Reviewers.docx [file pone.0248320.s005.docx]

When submitting your revision, we need you to address these additional requirements.

Responses to Reviewer comments in blue.

Line numbers in the revised manuscript without track changes in green.

1. Please ensure that your manuscript meets PLOS ONE's style requirements, including those for file naming. The PLOS ONE style templates can be found at:

https://journals.plos.org/plosone/s/file?id=wjVg/PLOSOne_formatting_sample_main_body.pdf

https://journals.plos.org/plosone/s/file?id=ba62/PLOSOne_formatting_sample_title_authors_affiliations.pdf

We checked again the style requirements and made the following changes:

L1-5 (L1-5): bold of the title and the short title has been removed

L10, 22 (L10, 22): add the city name to the affiliation

L19-25 (L19-25): Corrected the Affiliation.

L27-28 (L27-28): Corresponding author format has been changed to the journal requirements

L144 (L142): “Material and methods” Capital only the first word

L243-244 (L239): “Fig 2 and 3” citation of multiple figures

L322-324 (L313-315): Fig 4 caption has been moved below the table, since the table has first been cited.

L321 (L312): Table legend has been moved below the table.

L466 (L456): “Conclusion” 16pt font size as subheading of the discussion section

L695-712 (L683-692): Sort order of Supporting information in the order of the occurrence in the document.

L420 (L409): submitted related study has not been published yet, thus it was changed to “unpublished work”.

2. We note that you have stated that you will provide repository information for your data at acceptance. Should your manuscript be accepted for publication, we will hold it until you provide the relevant accession numbers or DOIs necessary to access your data. If you wish to make changes to your Data Availability statement, please describe these changes in your cover letter and we will update your Data Availability statement to reflect the information you provide.

The data DOI will be provided after the manuscript will be accepted for publication. However, as indicated in the cover letter, we meanwhile decided for another repository (iData Repository of iDiv (<https://idata.idiv.de/)>).

**Additional Editor Comments:**

Beside from comments from reviewers, please check:

There are some “effects” that should be replaced by affect (e.g., L. 36, L. 72 and so on).

L47, 86 (L46, 84): The wrong effects were replaced by affects.

Results: Should the units of sediment mass (g) be referred to some area unit (each g m-2)?

L261-262, 306-308 (L256-257, L301-303): We specified it as (g patch^-1^) all over the result section and modified it in the methods.

Please check the letters included in the Figures so as not to use same letters with different purposes (i.e., results of post-hoc tests and panel “order”.

Figure 4 (and Figure 5-7): We changed the letter indicating the panel order to a bold capital letter and added a small box around it. It makes the different purpose better visible. We additional explain it in the figure caption. We simultaneously changed the format of the letter for the panel order in the other result figure.

**Reviewers' comments:**

Review #1:

It would be relevant to add a paragraph in the discussion section related to the fact that the runs were conducted with the same water height. Changes in the hydrogeomorphological parameters (sediment supply and texture, channel slope, discharge) may affect vegetation effects on sediment trapping. Plant structural diversity could possibly explain sediment trapping variability only in a restricted range of discharge and sediment load. I believe you need to acknowledge that point.

L445-465 (L435-455): We agree, that abiotic parameters came a bit short in the discussion. We therefore now added a paragraph about the “Abiotic conditions” in the Discussion.

Specific comments
L. 36. ‘vegetation biomass positively effects’. Replace ‘effects’ by ‘affects’

L47, 86 (L46, 84): The verb “effect” was replaced by “affect”.

L. 44-45. ‘Thus, floodplains are among the most threatened ecosystems worldwide’ The causal linkage with fine sediment dynamics is not obvious. Clarifications are required about why floodplains are to be considered as threatened ecosystems in relation with fine sediment load.

True, the count as “most threatened ecosystem worldwide” is not the key consequence of the sediment and nutrient load. Instead, human activities are the main threat for riparian systems. Therefore, we now restructured the text to clarify our reasoning. The sentence now appears a few lines further down in the text.

L61-64 (L59-62): “However, human activities degraded river systems by straightening and embanking and thus reducing floodplain areas and their connectivity to the river (13,17). Today, floodplains are among the most threatened ecosystems worldwide (17, 18).”

L. 46. How does urbanization of floodplains increase erosion?

L56-58 (L54-56): ”…urbanization (through increased surface water run-off on sealed surfaces) of floodplains increase erosion,…”.

L58 (L57): We additionally added another reference (Hogg & Norris 1991 (12)).

L. 71. ‘Plant functional groups’ require a definition from the classical functional trait-based approach (e.g. Garnier, Lavorel, Diaz papers).

L86 (L84): Two references has been included: 36: Lavorel et al. 1997 and 37: Diaz et al. 2001.

L. 75. ‘due to natural variation’. What is the natural variation? Variation of what (genotypic, phenotypic, phenology, habitat conditions…)?

L89-90 (L87-88): We specified the term and change the sentence to: “… there is a wide variation of structural properties due to natural inter- and intraspecific variation, species sorting and grassland management.

L. 85-86. ‘On the other hand, dense vegetation can cause vertical mixing of the flow and increase turbulence that may change sedimentation patterns’. A vegetation matt with intermediate density can also enhance turbulence and erosion by causing flow divergence around the structures.

L99-100 (L97-98): True, it is not only the dense vegetation causing this sedimentation pattern; also intermediate density can cause that. We changed the sentence to: “On the other hand, increasing vegetation density can cause vertical mixing of the flow and increase turbulences that may change sedimentation patterns,…”

L. 105-106. What here is the exact meaning of ‘functionally more diverse vegetation patches (e.g. mixtures of functionally different plant species)’. Functionality (related to sediment trapping? Or to other functions such as photosynthesis if your consider leaf area…) needs to be better defined from the beginning.

L119-121 (L117-119): We changed both terms “functionally” to “structurally” in this sentence, because this term more precisely makes the point of the referenced study.
New: “However, another experiment found that structurally more diverse vegetation patches (e.g. mixtures of structurally different plant species) did not increase sedimentation significantly compared to monocultures (51).”

L. 112-113. ‘However, we expect that leaf surface structure has no strong effect on sedimentation on the soil surface underneath the vegetation.’ Why? You need to provide a short explanation. Large leafs could increase biomass and thus water blockage effect? But indeed leaf pubescence should not affect underneath sedimentation.

L126-128 (L124-126): We extended the sentence with a short explanation and added a reference. “However, we expect that leaf surface structure has no strong effect on sedimentation on the soil surface underneath the vegetation, since leaves only change the flow velocity and cause turbulence close to the leaf surface (53 (Albayrak et al. 2014)).”

L. 336. Replace (Kretz et al. 2020) by the ref number.

L373 (L364): The reference was replaced by the ref number 52.

L. 357. Replace (Proulx et al. 2014) by ref number.

L394 (L384): The reference was replaced by the ref number 48.

Reviewer #2:

Comments per section:
Please check “affect” and “effect” in the text (L36; L72)

L47, 86 (L46, 84): The verb “effect” were replaced by “affect”.

Abstract
1. Please add 1-2 sentences on how the sedimentation was determined (as described in Sample processing)

L41-42 (L40-42): We added a sentence about it. “We quantified the sediment by washing it off the biomass and off the fleece, drying the sediment and weighing it“.

Introduction
2. L53-54: Add sediment and “nutrient transport”, as in L54 you refer to “overfertilization”

L65-67 (L63-66): The text now reads “The consequence is an unbalanced sediment and nutrient transport along the river which causes overfertilization of the water and silting up of sediments in river branches, oxbows and river mouths (19–23).”

Experimental set-up
3. L175: Why is it important to state the company that built the flume?

Since the flume is unique, it does not have any model or series information. The company name helps to identify this flume at the experimental hall of the Leichtweiß-Institute for Hydraulic Engineering and Water Resources. Additionally, if someone wants to conduct a similar study and has the intention to let a company construct such a flume, it is helpful to know, which company has constructed the one in the present study. Therefore, we would keep it in the flume description, although we would be happy to remove the company name if the editor prefers this.

4. L182: What do you mean by “roughen the flow” – that they acted as a flow straightener to suppress secondary currents and establish uniform flow conditions? Please revise.

L207-210 (L205-208): We revised the sentence: “The first 5 m of the flume bed (inlet section) was covered with artificial lawn and bricks to roughen the flow bed and ensure fully turbulent flow conditions and a uniform flow field across the flume width upstream of the first line of vegetation patches.”

5. L183 and in the text: Please refer to “upstream and downstream” and not “in front and behind”

L210, 237, 240, 242 (L208, 233, 236, 238): “In front” was changed to “upstream”.

L241, 242, 410 (L237, 238, 400): “Behind” was changed to “downstream”.

6. L185: under dry conditions instead of at?

The sentence was changed to:
L211-214 (L208-211): “The vegetated flume section was separated by two closing walls and drained by a mobile submerged pump to enable removal of patches without distorting the sediment by lifting the patches through the whole water column.”

7. It is not clear to me how the water and the sediment / clay was mixed and where.

We mixed the water once with sediment to simulate a sediment-rich flood water. This sediment-rich water recirculated along the whole experiment. Every day we remobilized the sediment that settled on the ground with a scrubber. We improved the section about it in the following way:
L203-205 (L201-203): “The whole waterbody of 28 000 l was mixed with the sediment by adding clay and silt before the experiment started.”
L245-247 (L240-242): “After removing the patches, we opened the walls again and the sediment settled on the ground was remobilized using a scrubber.”

Was there a recirculating pump and the mixture of water and sediment was flowing through the test section with the respective discharge?

We had a closed water cycle. We improved the sentence in the following way:
L195-197 (L193-195): “For the experiment, we used a closed water cycle powered by a pump recirculating the water and the dissolved sediment with a constant discharge.”

Did the sediment settle upstream of the test section?

Some sediment settled on the ground of the flume, that is why it was remobilized with a scrubber after every run, to have it comparable between the runs.
L245-247 (L240-242): “After removing the patches, we opened the walls again and the sediment settled on the ground was remobilized using a scrubber.” (see also above)

Did you add the sediment prior to every run?

We hope that with the changes above we were able to clearly explain the procedure. We used the same water-sediment mixture all along the experiment in the same flume, but we remobilized sediment from the ground every day (after/prior to every run). For more details, see also our reply to the previous two comments, which dealt with the same issue.

It would be helpful to add some more information to the text and also a test program. It is not so clear to me how many runs were conducted.

We added a sentence with the number of runs at the end of the section “Experimental procedure”:
L230-232 (L226-228): “In total, we conducted 9 runs, 8 of them with each 12 vegetation patches and 4 control patches and one additional run with 16 control patches.”

8. Regarding the flow depth: Have you considered performing flume experiments under non-submerged, so emergent conditions or varying the discharge? Did you choose the discharge and flow depth based on the flume capacity? Why were these parameters not varied / discussed?

We agree that there are many hydrological parameters, that can potentially alter the sedimentation patterns and which are highly interesting in the given experimental setting. However, since we focused on investigating the vegetation characteristics, we needed to have the hydrological parameters comparable during the whole experiment.

L214-216 (L211-212): “With the overall constant hydraulic conditions we were able to focus on the relative differences in the structural characteristics of the vegetation during inundation.”

Nevertheless, some pre-studies were done and some a-priori decision were taken, and we agree that these were not sufficiently communicated. Thus, we added it in the section “Experimental set-up”:

L197-201 (L195-199): “Based on the results from pre-experiments, the technical limitations of the flume and the expert knowledge of hydraulic engineers, we decided for a constant discharge of 24-25 l sec-1, to be able to keep the balance between a low discharge simulating real floodplain inundation and a discharge high enough to keep the clay and silt floating in the flume water.”

The water depth was chosen with considering the flume capacity and the experimental set-up. To be able to pump all the water from the vegetated flume section to the upstream and downstream flume section, 45 cm water depth was the maximum that was possible within the 80 cm deep flume. We indicated this in the sentence:
L202 (L200): “… water depth of 45 cm (limited by the experimental set-up), so that all patches…”

L445-465 (L435-455): In addition, we now also added a paragraph in the discussion to discuss our choices of abiotic conditions, including submerged vs emerged conditions.

Experiment conduction (I recommend Experimental procedure)

L221 (L217): Changed the section heading to Experimental procedure

Results
9. L273: The variation of the flow velocity is similar to the mean value. Why? Was it due to the measurement device?

We measured the flow velocity to control for the positions in the flume. The observed differences varied indeed quite strongly, however no clear effect of the position (beside the higher velocity of the first patch line) was observed. Flow velocity is a complex parameter to measure, due to its complex spatial pattern and every device to measure flow velocity is highly sensitive to changes of flow direction and turbulences, especially at the relatively low flow velocity we used in the experiment.

Discussion
10. Similar to comment 8, it would be interesting to discuss the tested flow conditions (discharge, flow depth / submergence level) and characteristics of sediment and clay particles in the flow – and how these parameters affect the sedimentation processes. The flow velocity and particle size will affect the sedimentation processes, so more information on how you chose them would be interesting – also with respect to how to upscale them to prototype conditions.

L445-465 (L435-455): We now added a paragraph on “Abiotic conditions” to the Discussion section, in which we discuss a.o. the (possible) effects of flow conditions and the characteristics of sediment and clay particles.

We also improved the introduction and the methods section, regarding the link between fine sedimentation and nutrient retention. Fine sediment (silt and clay) is also the only grain size that we expect to be trapped on the vegetation biomass, due to its light weight compared to sand and coarser sediment. See the following alterations:

L67-70 (L66-68): “To improve the capacity of floodplains to retain sediment, and bound to it nutrients, it is crucial to obtain a holistic picture of the mechanisms optimizing fine sediment retention on natural floodplains”.

L 70-74 (L68-72): “Sedimentation can be described as a complex mixture of different biogeomorphologic processes, where structural properties, such as the density or height of the floodplain vegetation, are important for deposition of fine sedimentation and the morphology of the floodplain is crucial for deposition of coarse sediment (24,25).”

L 205-207 (L203-205): “We decided for a mixture of clay and silt, since the small grain sizes are important for associated nutrient retention and additionally we do not expect courser sediment to settle on the vegetation, due to their heavier weight.”

Figures
11. Fig. 3: What does a – b – b – b stand for above the box plot? It looks similar to the indication of the subfigures; please adapt

Fig 4: The letters a-b-b-b stand for groups that differ significantly from each other. We changed the letter indicating the panel order to a bold capital letter and added a small box around it. It makes the different purpose better visible. We now additionally explain what these letters stand for in the figure caption. We simultaneously changed the format of the letter for the panel order in the other result tables.

12. Fig. 5-6: I recommend starting with 0 at the x-axis

Fig 6-7 has been changed to start with 0 at the x-axis.

13. Fig. S1: I highly recommend including this figure in the main text, as it nicely illustrates what the experiments looked like

L174/178/180-183/225/229/234 (L172/176/178-181/221/225/230): Fig S1 was included in the main text as Fig 2. As a result, all later figures now have a changed number.
